# Molecular epidemiology of *bla*_CTX-M_ gene-producing uropathogenic *Escherichia coli* among Iranian kidney transplant patients: clonal dissemination of CC131 and CC10

**DOI:** 10.1186/s12941-021-00470-7

**Published:** 2021-09-08

**Authors:** Mehrdad Halaji, Shahrzad Shahidi, Behrooz Ataei, Abdolamir Atapour, Awat Feizi, Seyed Asghar Havaei

**Affiliations:** 1 Infectious Diseases and Tropical Medicine Research Center, Babol University of Medical Sciences, Babol, Iran; 2 Isfahan Kidney Diseases Research Center, Isfahan University of Medical Sciences, Isfahan, Iran; 3 Nosocomial Infection Research Center, Isfahan University of Medical Sciences, Isfahan, Iran; 4 Department of Biostatistics and Epidemiology, School of Health, Isfahan University of Medical Sciences, Isfahan, Iran; 5 Department of Microbiology, School of Medicine, Isfahan University of Medical Sciences, Isfahan, Iran

**Keywords:** Uropathogenic *Escherichia coli*, CTX-M, MLST, Virulence factor, Phylogenetic group

## Abstract

**Background:**

This study aimed to investigate the phylogenetic characterization and virulence traits of uropathogenic *Escherichia coli* (UPEC) isolated from kidney transplant patients (KTPs) as well as non-KTPs and analyze the clonal distribution of Extended spectrum β-lactamases (ESBLs)-producing UPEC containing *bla*_CTX-M_ gene.

**Methods:**

To this end, we determined virulence marker and the phylogenetic characterization of UPEC in non-KTPs (n = 65) and KTPs (n = 46). The non-KTPs were considered the control group of the study. Also, according to the Achtman scheme, we performed multilocus sequence typing to assess the relationship between twenty-nine of ESBL-producing isolates containing *bla*_CTX-M_ gene.

**Results:**

According to the results of PCR assay, the prevalence of virulence factor genes ranged from 0% (*cnf* and *papG* III) to 93.7% (*fimH*). Also, KTP isolates significantly differed from non-KTP isolates only in terms of the prevalence of *pap GI* elements. Moreover, the most frequent UPEC isolates were in phylogenetic group B_2_, followed by group D (18.9%), and group A (13.5%). Furthermore, except for phylogenetic group C, there was no significant correlation between phylogenetic distribution in KTPs and non-KTPs. Additionally, MLST analysis of bla_*CTX-M*_ carrying isolates identified 18 unique sequence types (ST) the most common of which was ST131 (24.1%), followed by ST1193 (10.3%), while fourteen STs were detected only once.

**Conclusions:**

The results further revealed significant differences between the UPEC isolates from KTPs and non-KTPs regarding the phylogroups C and *PAI* gene. Based on MLST analysis, we also observed a relatively high diversity in UPEC isolates obtained from KTPs and non-KTPs. Moreover, clonal complex (CC) 131 and ST131 were found to be the most prevalent clones and ST types, respectively. Besides, for the first time, ST8503 were reported in KTPs. These results suggested regular studies on characterization of UPEC isolates among KTPs.

**Supplementary Information:**

The online version contains supplementary material available at 10.1186/s12941-021-00470-7.

## Background

Recently, in kidney transplant patients (KTPs), urinary tract infections (UTI) have been complicated by the widespread disseminated bacterial agents, particularly *Escherichia coli*, which are resistant to first-line antimicrobial options [1, 2]. Uropathogenic *Escherichia coli* (UPEC) are common human pathogens associated with both nosocomial and community-acquired UTI [3].

The worldwide increasing extended-spectrum β-lactamases (ESBL) and multi-drug resistance (MDR) of *E. coli* strains are responsible for a high proportion of UTI frequently affecting KTP [4]. Production of ESBL is one of the primary interfering mechanisms in resistance of *E. coli* to β-lactam antibiotics, including first- and third-generation cephalosporins. According to several reports, the most prevalent ESBL clone is related to the wide spreading of CTX-M, which has been replaced with *bla*_SHV_ and *bla*_TEM_, as the most prevalent ESBL genes [5–7].

The dissemination of CTX-M family among UPEC is mediated by the rapid spread of plasmids and mobile genetic elements. UTI with bla_*CTX-M-15*_-producing-UPEC in humans is a major concern especially in KTP [8].

Moreover, *E. coli* can be typed based on their O serotypes, phylogroups (A, B1, B2, C, D, E, and F), and sequence types (ST), which express different pattern of virulence factors (VFs) [9, 10]. Most UPEC strains infecting immunocompetent humans belong to phylogroups group B_2_ and, to a lesser extent, phylogroups D, which can overcome host defenses and invade host tissues in presence of VFs [10, 11].

Previous studies have also revealed that many UPEC isolates belong to a small number of clonal groups presenting a range of VFs and variable antibiotic resistance pattern. According to the literature, ST 131, ST69, ST73, and ST95 are pandemic UPEC clones. However, recently, other clones, such as ST10 and ST127 have emerged among UPEC strains [12–14]. ST131 containing CTX-15 enzyme is associated with high potential virulence and high level of antibiotic resistance (two major public health problems) [15].

Furthermore, since UTI caused by UPEC in KTPs is considered one of the most important post-transplantation complications increasing the risks of graft rejection, it is critical to determine the characterization and population structure of ESBL-producing UPEC [11].

In Iran, previous studies focused on the investigation of UPEC isolated from hospitalized patients in clinical samples [16–18]; thus, no sufficient data are available about the UPEC isolated from KTPs [19, 20]. Also, up until today, there is no reports about the STs of UPEC isolates that have distributed in Iran were available.

Accordingly, we planned to investigate the phylogenetic characterization and virulence traits of UPEC isolated from KTPs and non-KTPs with symptoms of UTI in Iran and analyze the clonal distribution of ESBL-producing UPEC containing CTX-M isolated from KTPs as well as non-KTPs isolates.

## Materials and methods

### Study design and setting

In this case–control study, UPEC isolates were collected from June 2019 to October 2019 from a total number of 111 patients, who were assigned to two groups of non-KTPs (n = 65) and KTPs (n = 46). All the patients had UTI symptoms. This study was performed in three laboratory centers as well as two nephrology private clinics affiliated to Isfahan University of Medical Sciences (IUMS) [21]. This study was also confirmed and permitted by the Ethics Committee of IUMS (IR.MUI.MED.REC.1398.202). It is necessary to mention that all the UPEC isolates were confirmed in previous our study [21].

### Antimicrobial susceptibility testing and phenotypic assays

Antimicrobial susceptibility testing was carried out using the disc diffusion method based on CLSI guidelines [21]. Based on CLSI recommendations, the double-disk synergy test (DDST) was used as a phenotypic method to detect ESBL-producing strains [21, 22].

### Molecular detection of resistance genes

The presence of ESBLs genes including *bla*_CTX-M_, *bla*_TEM_, *bla*_SHV_ were determined using PCR assays as previous described [23, 24]. The PCR amplicons of ESBL-producing isolates contain *bla*_CTX-M_ gene were sequenced, and the DNA sequence of each gene was assigned in the GenBank nucleotide database at http://www.ncbi.nlm.nih.gov/blast/.

### Virulence gene profiling

The presence of the most relevant UPEC genes were detected by PCR using a set of specific primers as previously described. They included adhesins (*papG* I, II, III, *sfaDE*, *afaBC*, *fimH*), siderophores (*iutA* and *chuA*), miscellaneous (*PAI*), and toxins (*hlyA*, and *cnf1*) [25, 26]. Moreover, the virulence score (VF score; number of VF genes) was considered as the sum of all VFs for each isolates. Positive controls for (*afa*, *fimH*, *hly A* and *iutA*) and (*pap G1*,* pap G III* and *chuA*) genes were obtained from previous study performed by Malekzadegan et al. and Hojabri et al., respectively [27, 28].

### Phylogenetic analysis and multilocus sequence typing (MLST)

Each isolate was assigned to one of the major *E. coli* phylogroups (A, B1, B2, C, D, E, and F) by quadruplex PCR as described previously [29]. MLST was performed to assess the twenty-nine of ESBL-producing isolates contain *bla*_CTX-M_ gene according to the Achtman scheme using amplification of the seven housekeeping genes including *adk*, *fumC*, *gyrB*, *icd*, *mdh*, *purA*, and *recA* available at MLST website (http://mlst.warwick.ac.uk/mlst/dbs/Ecoli). STs were assigned to clonal complex (CCs), single-locus variants (SLV), double-locus variants (DLV), and singletons using the goeBURST algorithm using PHYLOViZ 2.0 software [30].

### GenBank accession numbers

The nucleotide sequences of the *bla*_CTX-M_ gene have been assigned to GenBank under the following accession numbers:

MT274743, MN997127, MT007111, MT274741, MT274742, MT293606, MT293607, MT293608, MT293609, MT293610, MT293611, MT293612, MT310667, MT310668, MT310669, MT310670, MT310671, MT310672, MT310673, MT310674, MT310675, MT310676, MT310677, MT310678, MT310679, MT310680, MT501676, MT501677.

### Statistical analysis

Continuous and categorical data were reported as mean ± standard deviation (SD) and frequency (percentage). Categorical data were compared between groups by using chi-squared test and pairwise comparisons were conducted by adopting Bonferroni correction for multiple testing. Continuous variables (VF and antibiotic resistance scores) were compared between groups by using one-way Analysis Of Variance (ANOVA) and Gabriel post hoc test has been conducted for pirwise comparisons. All statistical analyses were performed using SPSS 16 (IBM Corp., USA). Statistical significance level was considered as *P* value of < 0.05.

## Results

According to the results of PCR assay, 90.9% of the UPEC isolates was positive for one or more of the VF genes (Table 1). Among all of the isolates, the prevalence of individual VF genes ranged from 0% (*cnf* and *papG* III) to 93.7% (*fimH*) (Additional file 1: Figure S1A–G).

**Table 1:** Distribution of genes encoding virulence factors among KTP and non-KTP isolates

Function	Virulence factor	Total (n = 111) No. (%)	KTP (n = 46) No. (%)	Non-KTP (n = 65) No. (%)	P-value
Miscellaneous	*PAI*	62 (55.9)	28 (60.9)	34 (52.3)	0.37
Adhesions	*Sfa*	54 (48.6)	24 (52.2)	30 (46.2)	0.53
	*Pap G I*	43 (38.7)	24 (52.2)	19 (29.2)	0.01
	*Pap G II*	–	–	–	–
	*Pap G III*	31 (27.9)	15 (32.6)	16 (24.6)	0.35
	*afa*	17 (15.3)	6 (13)	11 (16.9)	0.57
	*fim H*	104 (93.7)	43 (93.5)	61 (93.8)	0.93
Toxins	*hyl A*	9 (8.1)	2 (4.3)	7 (10.8)	0.3
	*cnf*	–	–	–	–
Siderophores	*iutA*	72 (64.9)	27 (58.7)	45 (69.2)	0.25
	*chuA*	80 (72.1)	34 (73.9)	46 (70.8)	0.71
VF score (mean, median, range)	–	4.82 ± 1.3, 5(2–9)	4.97 ± 1.2, 5 (2–8)	4.72 ± 1.35, 5 (2–9)	0.31
Phylogenetic groups	B2	43 (38.7)	18 (39.1)	25 (38.5)	0.94
	D	21 (18.9)	10 (21.7)	11 (16.9)	0.52
	A	15 (13.5)	9 (19.6)	6 (9.2)	0.1
	B1	10 (9)	3 (6.5)	7 (10.8)	0.51
	C	6 (5.4)	0	6 (9.2)	0.03
	E	5 (4.5)	1 (2.2)	4 (6.2)	0.31
	F	4 (3.6)	2 (4.3)	2 (3.1)	0.72
	Non	7 (6.3)	3 (6.5)	4 (6.2)	0.93

Values are mean ± SD for continuous and frequency (percentage) for categorical data. P-values resulted from Chi-Squared and ANOVA for categorical and continuous data, respectively

Also, KTP isolates significantly differed from non-KTP isolates only in terms of *pap GI* elements prevalence [52.2% (24/46) versus 29.2% (19/65), P = 0.01], not regarding any other studied virulence genes. In addition, the overall median aggregate VF scores were higher for KTP isolates (median VF score 4.97, range 2–8) than those for non-KTP isolates (median VF score 4.72, range 2–9). However, there was no significant correlation between VF score and type of patient. Further, the way how genes encoding VFs were distributed among KTP and non-KTP isolates is shown in Table 1

### Phylogenetic groups

According to the quadruplex PCR assay results, most (38.7%) of the UPES isolates belonged to phylogenetic group B_2_, followed by group D (18.9%), group A (13.5%), B1 (9%), C (5.4%), E (4.5%), and F (3.6%) (Additional file 1: Figure S2). The remaining 6.3% of the isolates were found to be un-typeable. Phylogenetic group B_2_, D, A, and F were also more prevalent in the KTPs than the non-KTPs isolates, while the phylogenetic group B_1_, C, and E were more frequent in control group. However, except for phylogenetic group C that was more significantly prevalent in control group, there was no significant difference between KTPs and non-KTPs regarding phylogenetic distribution. Distribution of the phylogenetic groups is summarized in Table 2.

**Table 2:** Distribution of VF genes according to phylogroups

Virulence factor	Phylogenetic group								P value
	B_2_ 43 No. (%)	D 21 No. (%)	A 15 No. (%)	B_1_ 10 No. (%)	C 6 No. (%)	E 5 No. (%)	F 4 No. (%)	Unknown 7 No. (%)	
*PAI*	37(86)	8 (38.1)	4 (26.7)	2 (20)	1 (16.7)	1 (20)	2 (50)	7 (100)	≤ 0.05
*sfa*	22 (51.2)	11 (52.4)	7 (46.7)	3 (30)	2 (33.3)	4 (80)	2 (50)	3 (42.9)	> 0.05
*pap G I*	16 (37.2)	8 (38.1)	9 (60)	6 (60)	–	2 (40)	1 (25)	1 (14.3)	> 0.05
*pap G II*	–	–	–	–	–	–	–	–	> 0.05
*pap G III*	11 (25.6)	6 (28.6)	7 (46.7)	3 (30)	1 (16.7)	–	–	3 (42.9)	> 0.05
*afa*	8 (18.6)	5 (23.8)	1 (6.7)	1 (10)	–	–	–	2 (28.6)	> 0.05
*fim H*	39 (90.7)	21 (100)	14 (93.3)	9 (90)	6 (100)	5 (100)	3 (75)	7 (100)	> 0.05
*hyl A*	6 (14)	2 (9.5)	–	1 (10)	–	–	–	–	> 0.05
*cnf*	–	–	–	–	–	–	–	–	> 0.05
*iutA*	31 (72.1)	13 (61.1)	9 (60)	5 (50)	4 (66.7)	3 (60)	3 (75)	4 (57.1)	> 0.05
VF score (mean, median, range)	4.95 ± 1.1, 5 (3–8)	5.5 ± 1.2, 6 (3–7)	4.4 ± 1.1, 4 (2–7)	4 ± 1.1, 4 (2–6)	3.3 ± 1, 4 (2–4)	5 ± 0.7, 5 (4–6)	3.7 ± 0.95		0.01^a^

Values are mean** ± **SD for continuous and frequency (percentage) for categorical data. P-values resulted from Chi-Squared and ANOVA for categorical and continuous data, respectively

^a^Distribution of VF genes in phylogroups B_2_ was significantly more than phylogroups C

### Correlations between phylogroups and VAGs

According to distribution of VFs in phylogroups, *fimH*, *PAI*, and *iutA* were more frequent among B_2_ members followed by *fimH*, *iutA*, and *sfa*, which were more frequent among phylogroups D and *fimH*, *papGI* and *iutA* more common among phylogroups A and B1 members. Additionally, *sfa* were more prevalent in phylogroup C than other groups. Frequency of VF genes based on phylogroups is shown in more details in Table 2.

Furthermore, phylogenetic groups considerably varied in VF content so that the highest median VF scores (and ranges) were 6 (3–7) for isolates belonged to phylogroup D, followed by phylogroup B_2_, E with 5 (3–8), and 5 (4–6), respectively, while the lowest median VF score (and range) were 3.3 (2–4) for isolates belonged to phylogroup C. Statistical analysis also showed no significant differences between VF score and various phylogroups, except for phylogroup B_2_ and C. Moreover, among different phylogroups, *pai* gene was statistically significant in phylogroup B_2_ in comparison to other phylogroups.

CTX-M group (mainly CTX-M-15) was also found to be more prevalent in B_2_, D, and A phylogroups, while *bla*_TEM_ was more frequent in D, F, and A phylogroups.

Phylogroups further varied in antibiotic susceptibility pattern; the most susceptible phylogroup was phylogroups C (resistance score median 2 and range 0–8), and the most resistant phylogroup was phylogroup B_1_ (median 6 and range 0–11), followed by phylogroups D and B_2_ (median 6 and range 0–11). Overall, we noticed that 35% of the UPEC strains were ESBL-producer [21]. Isolates belonged to phylogroups F and B_2_ exhibited a high prevalence (83.3%) of ESBL. Distribution of the antimicrobial resistance profiles according to phylogroups is also shown in Table 3.

**Table 3:** Distribution of the antimicrobial resistance profiles according to phylogroups

Antibiotics	B_2_ 43 No. (%)	D 21 No. (%)	A 15 No. (%)	B_1_ 10 No. (%)	C 6 No. (%)	E 5 No. (%)	F 4 No. (%)	Unknown 7 No. (%)	P value
Amoxicillin/clavulanic	14 (32.6)	10 (47.7)	5 (33.4)	4 (40)	0	0	1 (25)	2 (28.6)	> 0.05
Piperacillin–tazobactam	4 (9.3)	3 (14.3)	1 (6.7)	3 (30)	0	0	1 (25)	2 (28.6)	> 0.05
Cefoxitin	10 (23.3)	4 (19)	4 (26.7)	2 (20)	2 (33.3)	2 (40)	1 (25)	0	> 0.05
Ceftazidim	19 (44.2)	9 (42.9)	6(40)	5 (50)	2 (33.3)	1 (20)	2 (50)	1 (14.3)	> 0.05
Cefixim	20 (46.5)	13 (61.9)	5 (33.3)	5 (50)	2 (33.3)	0	1 (50)	3 (42.9)	> 0.05
Cefepime	12 (27.9)	3 (14.3)	4 (16.6)	2 (20)	5 (83.3)	0	2 (50)	1 (14.3)	> 0.05
Imipenem	0	0	0	0	0	0	0	0	> 0.05
Meropenem	0	0	0	0	0	0	0	0	> 0.05
Trimethoprim/sulfamethoxazole	24 (55.8)	13 (61.9)	9 (60)	8 (80)	4 (66.7)	4 (80)	3 (75)	5 (71.4)	> 0.05
Ciprofloxacin	22 (51.2)	8(38.1)	7 (46.7)	6 (60)	1 (16.7)	1 (20)	3 (75)	3 (42.9)	> 0.05
Ofloxacin	24 (55.8)	9 (42.9)	6 (40)	6 (60)	2 (33.3)	3 (60)	3 (75)	3 (42.9)	> 0.05
Nalidixic acid	32 (74.4)	15 (71.4)	10 (66.7)	8 (80)	3 (50)	3(60)	4 (100)	3 (42.9)	> 0.05
Norfloxacin	21 (48.8)	8 (38.1)	7 (46.7)	6 (60)	1 (16.7)	1 (20)	2 (50)	3 (42.9)	> 0.05
Nitrofurantoin	3 (7)	7 (33.3)	4 (26.7)	2 (20)	1 (16.7)	2 (40)	1 (25)	0	≤ 0.05
Gentamicin	12 (27.9)	(14.3) 3	4 (26.7)	3 (30)	1 (16.7)	3 (60)	0	0	> 0.05
Amikacin	0	0	0	1 (10)	0	0	0	1 (14.3)	> 0.05
Resistance score (mean, median, range)	4.88, 5, (0–11)	4.9±, 5, (0–11)	4.33, 3, (0–11)	5.5, 6, (0–11)	2.83, 2, (0–8)	4, 4, (0–8)	5.5, 5, (0–10)	3.7, 3, (0–9)	> 0.05
ESBL	17(39.5)	7 (33.3)	4 (26.6)	2 (20)	2 (33.3)	1 (20)	2 (50)	–	> 0.05
*CTX-M*	25 (58.1)	11 (52.4)	8 (53.3)	3 (30)	3 (50)	2 (40)	1 (25)	6 (85.7)	> 0.05
*TEM*	20 (46.5)	12 (57.1)	7 (46.7)	2 (20)	2 (33.3)	2 (40)	2 (50)	3 (42.9)	> 0.05
*SHV*	1 (2.3)	2 (9.5)	1 (6.7)	1 (10)	–	–	–	1 (14.3)	> 0.05

Among 31/5% (35/111) of ESBL-producing isolates, 82.9% (29/35) contained bla_*CTX-M*_ genes. Of these bla_*CTX-M*_ positive isolates, 27 isolates carried bla_*CTX-M-15*_, and 2 isolates carried bla_*CTX-M-55.*_

**Fig. 1:**
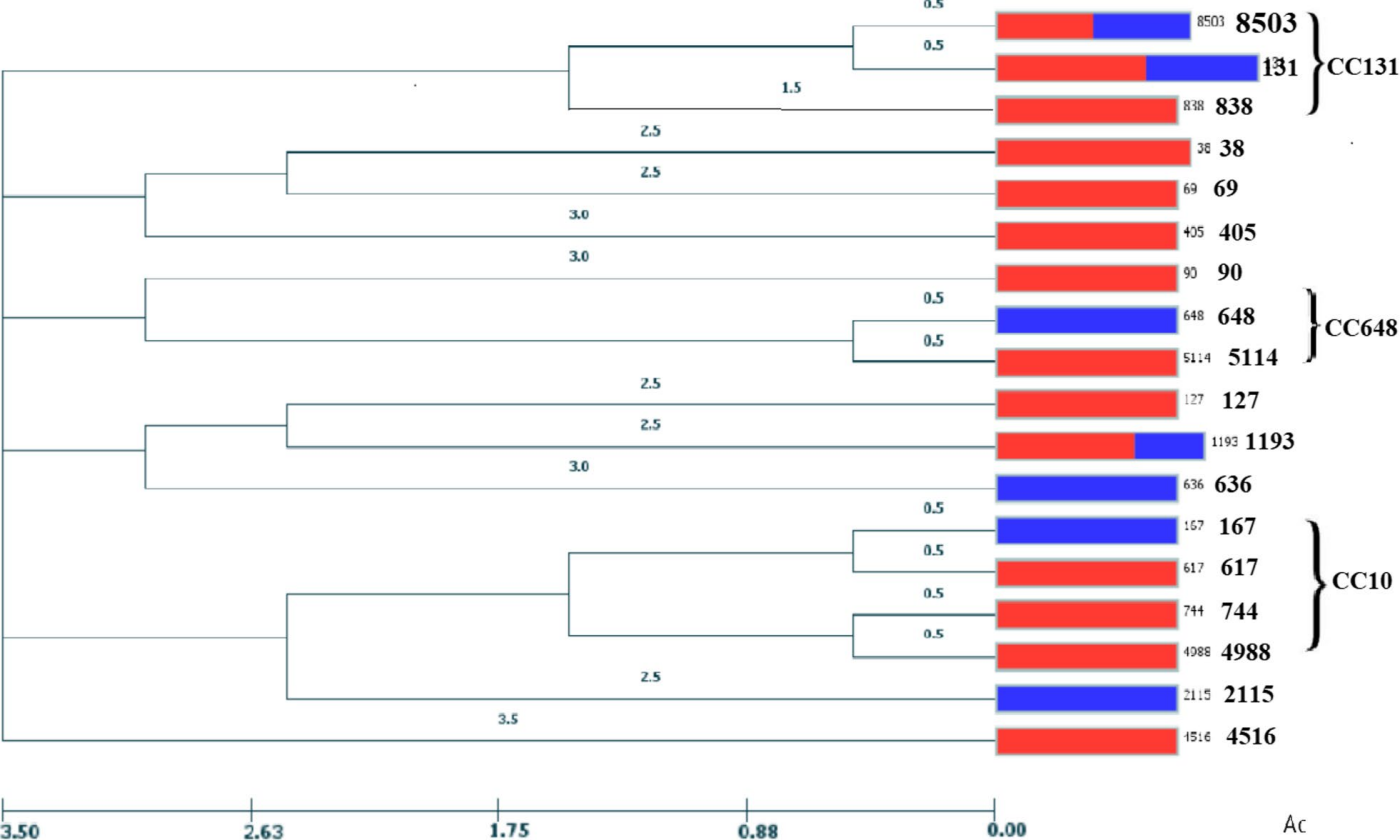
Phylogenetic tree of MLST for the bla_*CTX-M*_ positive strains; clonal complexes are shown with haloes. The colors of the circles correspond to the type of patients (Red: transplanted patients; Blue: Control group)

MLST analysis of bla_*CTX-M*_ carrying isolates identified 18 unique STs, the most common were ST131 (7; 24.1%) followed by, ST1193 (3; 10.3%), ST38 (2; 6.9%), ST8503 (2; 6.9%) and fourteen STs were detected only once ST69, ST90, ST167, ST127, ST405, ST617, ST636, ST648, ST744, ST838, ST2115, ST4516, ST4988 and ST5114. Overall, using the goeBURST algorithm, STs were belonged to eight CCs including CC131 (ST131, 8503 and 838), CC10 (ST167, 744, 4988 and 617), C14 (ST1193), CC23 (ST90), CC38 (ST38), CC69 (ST69), CC405 (ST405) and CC648 (ST648) and remaining STs were classified as singleton. The distribution of STs and characteristics of the 29 bla_*CTX-M*_-producing UPEC is shown in Table 4. Moreover, phylogenic analysis of our STs in each CCs has been shown in Fig. 1.

**Table 4:** Characteristics of the 29 bla_*CTX-M*_ -producing UPEC

Strain	Type of patients	Antibiotic resistance pattern	ESBL genes	ESBL	Virulence profile	Phylogenetic groups	ST	CC
103	KTP	TS, NA, Nor, OFX, CIP, CAZ, CFM, AUG	bla_*CTX-M-15*_	+	*fimH*, *PAI*, *sfa*, *afa*	D	90	23
120	KTP	TS, NA, Nor, FOX, OFX, GM, FEP CIP, CAZ, CFM, AUG	bla_*CTX-M-15*_, bla_*TEM*_	+	*fimH*, *PAI*, *sfa*, *iut*, *papG1*	D	405	405
122	KTP	TS, TZP, CAZ, CFM, FM	bla_*CTX-M-15*_ bla_*TEM*_	+	*fimH*, *papG1*, *papG3*	D	69	69
9	KTP	TS, NA, Nor, FOX, OFX, GM, FEP CIP, CAZ	bla_*CTX-M-15*_	+	*fimH*, *PAI*, *iut*	B_2_	131	131
127	KTP	TS, NA, Nor, FOX, OFX, CIPFEP, TZP, CAZ, CFM, AUG	bla_*CTX-M-15*_, bla_*TEM*_	+	*fimH*, *PAI*, *iut*, *afa*, *papG1*	B_2_	8503	131
147	KTP	TS, NA, Nor, OFX, CIP, CAZ, CFM, AUG	bla_*CTX-M-15*_, bla_*TEM*_	+	*fimH*, *PAI*, *iut*	B_2_	2114	648
112	KTP	TS, NA, Nor, FOX OFX, CIP, GM CAZ CFM,	bla_*CTX-M-15*_	+	*fimH*, *PAI*, *iut*	B_2_	1193	14
124	KTP	TS, NA, Nor, FOX OFX, CIP, GM CAZ, CFM	bla_*CTX-M-15*_	+	*fimH*, *PAI*, *sfa*, *iut*, *papG3*	B_2_	1193	14
138	KTP	TS, NA, FEP, CAZ, CFM	bla_*CTX-M-15*_, bla_*TEM*_, bla_*SHV*_	+	*fimH*, *sfa*, *iut*, *afa*, *papG3*	D	38	38
72	KTP	TS, NA, Nor, OFX, CIP, CAZ, CFM	bla_*CTX-M-15*_	+	*fimH*, *sfa*, *iut*	F	38	38
139	KTP	TS, NA, Nor, OFX, CIP, GM, FEP, TZP, CFM, FM	bla_*CTX-M*_*-*_*55*_	+	*fimH*, *PAI*, *sfa*, *iut*	B_1_	617	10
121	KTP	TS, NA, Nor, FOX, OFX, GM, CIP, CAZ CFM	bla_*CTX-M-55*_,* bla*_*TEM*_	+	*fimH*, *sfa*, *iut*, *papG1*	A	744	10
101	KTP	NA, NOR, OFX, CIP, FEP, AUG	bla_*CTX-M-15*_	+	*fimH*, *PAI*, *sfa*, *iut*, *afa*, *hly*, *papG1*	B_2_	838	131
128	KTP	TS, NA, Nor, OFX, GM, CIP, CAZ	bla_*CTX-M-15*_, bla_*TEM*_	+	*fimH*, *papG1*, *papG3*	A	4988	10
110	KTP	TS, NA, NOR, OFX, GM, CIP, FEP, TZP, CAZ, CFM	bla_*CTX-M-15*_, bla_*TEM*_	+	*fimH*, *sfa*, *papG3*	B_2_	4516	
146	KTP	TS, NA, Nor, OFX, CIP, CAZ	bla_*CTX-M-15*_, bla_*TEM*_	+	*fimH*, *PAI*, *iut*	B_2_	131	131
148	KTP	TS, NA, Nor, OFX, GM, CIP, CAZ, FEP	bla_*CTX-M-15*_, bla_*TEM*_	+	*fimH*, *PAI*, *sfa*, *iut*	B_2_	131	131
129	KTP	TS, NA, Nor, FOX, OFX, CIP, TZP, FEP, CAZCFM, AUG	bla_*CTX-M-15*_, bla_*TEM*_	+	*fimH*, *papG1*, *papG3*	A	131	131
137	KTP	NA, GM, TZP, CAZ, CFM, FM	bla_*CTX-M-15*_, bla_*TEM*_	+	*fimH*, *PAI*, *sfa*, *iut*	D	127	127
57	Non-KTP	NA, Nor, Fox, OFX, GM, CIP, CAZ, FM	bla_*CTX-M-15*_	+	*fimH*, *PAI*, *sfa*, *afa*, *papG1*, *papG3*	B_2_	8503	131
89	Non-KTP	TS, NA, NOR, OFX, CIP, CAZ, CTX, CFM, AUG	bla_*CTX-M-15*_	+	*fimH*, *papG1*	B_2_	131	131
61	Non-KTP	TS, NA, OFX, CAZ, CFM	bla_*CTX-M-15*_	+	*fimH*, *PAI*, *iut*, *papG3*	A	2115	
78	Non-KTP	NA, Nor, Fox, OFX, GM, CIP, CAZ, CFM, AUG	bla_*CTX-M-15*_	+	*fimH*, *PAI*, *iut*, *papG1*	B2	1193	14
59	Non-KTP	TS, NA, Nor, Fox, OFX, GM, FEP CIP, CAZ, CFM	bla_*CTX-M-15*_	+	*fimH*, *PAI*, *sfa*, *iut*	B_2_	131	131
80	Non-KTP	TS, NA, TZP, CAZ, CAZ, CFM, AUG	bla_*CTX-M-15*_,* bla*_*TEM*_	+	*fimH*, *PAI*, *iut*, *afa*, *papG1*	B_2_	636	–
87	Non-KTP	TS, OFX, GM, CAZ	bla_*CTX-M-15*_,* bla*_*TEM*_	+	*PAI*, *sfa*	E	–	–
56	Non-KTP	NA, Nor, OFX, CIP, CAZ, CFM	bla_*CTX-M-15*_	+	*fimH*, *PAI*, *sfa*, *afa*, *papG1*	B_2_	648	648
33	Non-KTP	TS, CAZ, CFM	bla_*CTX-M-15*_	+	*iut*, *afa*	B_2_	167	10
79	Non-KTP	TS, NA, Nor, OFX, GM, FEP CIP, CAZ, CFM	bla_*CTX-M-15*_	+	*fimH*, *PAI*, *sfa*, *iut*	B_2_	131	131

## Discussion

In this case–control study, we characterized the virulence potential, phylogenetic group distributions, and clonal diversity in KTPs and non-KTPs with UTI caused by UPEC. To date, only some studies have been carried out on the above-mentioned items in KTPs. Therefore, this is the first study to simultaneously investigate these aspects among KTPs in Iran [19, 20] to assist physicians to conform with the latest pathogenic strains causing UTI by having a better perception of the virulence markers of UPEC strains, especially in transplant patients.

UPEC isolates produce different types of virulence factor, such as adhesion, siderophores and toxins, which are vital for attaching to, colonizing, and invading their host. Of VFs, genes encoding adhesions such as type‐1 fimbriae, which are essential for establishment and progression of UTI, are the most common occurring ones in UPEC isolates. As we expected, the results revealed that *fimH* was the most frequent virulence genes followed by *sfa* and *papG I*. These findings are in consistent with results of other studies indicated that *fimH* is the most predominant virulence factor between UPEC isolates [16, 27, 31–34].

In agreement with a study conducted in another part of Iran (79.4%) as well as in South Korea (100%), in the present study, the *sfa* gene was the second most common adhesion gene (48.6%) among the studied isolates [27, 35].

In several other studies performed on KTPs, the *sfa* was reported to include 26.1% [20], 19% [36], and 0% [34] of UPEC isolates. All of these percentages are lower than what we detected in the current work.

In this study, the second most common VF gene was found to be *iutA*. *iutA* is the siderophore-mediated uptake systems in UPEC strain that contribute to resistance to serum killing. As shown by findings of the current work, 64.9% of UPEC was PCR-positive for *iutA*. These results are broadly consistent with those reported considering the KTP [34], community [14], and hospitalized population [27].

In the presents study, although KTP isolates had higher VFs score than non-KTP isolates, we did not find significant difference between the VF scores of KTP and non-KTP isolates. However, among adhesion factors, *pap G1* was significantly more frequent in KTP isolates than non-KTP isolates (*p* = 0.01). Distribution of VFs is usually varied in UPEC. This discrepancy could be due to the difference in disease type, geographic area, and the studied population. Based on phylogenetic analysis, *E*. *coli* pathotypes have been classified into four main phylogenetic groups of A, B1, B_2_, and D. According to previous studies, B_2_ and D are the most frequent phylogroups among UPEC isolates, whereas phylogroups A and B_1_ have been mostly recognized as commensal *E*. *coli* isolates [35, 37].

This study also revealed that the major phylogenetic group in KTP isolates was B_2_ (39.1%), followed by D (21.7%), and A (19.6%), while the predominance of phylogenetic groups in non-KTP isolates were B_2_ (38.7%), followed by D (18.9%), and B_1_ (9%). However, there was no significant difference between phylogenetic dissemination in KTPs and non-KTPs, except for phylogenetic group C that was significantly predominant in the control group. These findings are in agreement with several other studies, which indicated that the majority of UPEC strains, with an increased potential to cause UTIs, belonged to group B_2_ and D [31], while diarrheagenic or commensals strains often belonged to phylogroups A and B_1_ [38].

Furthermore, it should be pointed out that this study’s findings suggested that UTIs can have extraintestinal origin even in immunosuppressed patients such as KTPs.

In 2019, Basha et al. described the phylogroup frequency of UPEC strains in KTP whereby the most common groups were B_2_, A, and B_1_, while in control group, B_2_, B_1_, and D were the most common groups. These findings strongly support the present work’s reports [39]. Similarly, Tashk et al. revealed that the majority of UPEC isolates from KTPs belonged to phylogroups A, B_1_, and F, which is uncommon for UPEC strain [40].

Unlike the current study, Khairy et al. reported that the predominant phylogenetic groups among UPEC isolates were groups A, followed by B_2_ and D [41]. Also, in another study conducted by Farajzadah Sheikh et al., phylogroup D (58%) was detected as the most common phylogroups of UPEC strains [16]. These discrepancies suggested that distribution of phylogenetic groups might vary across different regions, features of host, environmental, social, and geographic conditions.

In another part of our study, finding revealed that phylogroup B_1_, followed by phylogroups D and B_2_ had high level of resistance score. On the other hand, UPEC isolates belonged to phylogroups F and B_2_ exhibited a high frequency of ESBL strain. According to previous report, isolates belonged to phylogenetic group B_2_ and D had high level of antibiotic resistance compared to other groups, which our results in partially in agreement with their results [33, 42].

Besides, resistant rate of nitrofurantoin among different phylogenetic group was significant and member of phylogroups E and D had high level of resistance than other phylogroups. However, due to the appropriate level of sensitivity to nitrofurantoin compared to other oral antibiotics, nitrofurantoin can be recommended as a suitable oral drug to be prescribed by physicians to these patients.

Moreover, member of phylogroups B_2_ and D as most important phylogroups, had high level of sensitivity to carbapenems, piperacillin–tazobactam and amikacin.

Therefore, based on previous findings and our results, the type of phylogenetic group and the pattern of antibiotic resistance associated with it may be appropriate and useful in the correct administration of antibiotics, especially in patients with kidney transplant.

In the present study, according to the observed population structure of *E. coli*, members of CC131 predominantly belonged to phylogenetic group B_2_. By the widely used quadruplex PCR method, we further confirmed that 90% of members of CC131 found in this study could be classified into B_2_ phylogenetic group.

Among the ESBL-producing UPEC isolates, *bla*_CTX-M-15_ is mostly associated with the ST131 clonal group, which has emerged in many parts of the world since 2000 [42]. Our results confirmed studies reported *bla*_CTX-M-15_ as the dominant CTX-M type among *E. coli* ST131 isolates in western Asia (68%) [43], Iran [44], and Spain (95%) [45].

On the other hand, a recent meta-analysis study described that ST131 clone occurrence among ESBL-producing *E. coli* was 42.7%, which is partially comparable with this work’s results with 34.5%. The observed discrepancies among the studies’ results were mostly related to different sample sizes, studies’ population, or detected O-serogroups.

We further perceived that ST8503 as a member of CC131 belonging to phylogroup B_2_ co-harbored *bla*_CTX-M_, and *bla*_TEM_ genes. ST8503 as an MDR ESBL-producing ST having high pathogenicity had not previously been reported from KTPs. So, our results confirmed the emergence of *bla*_CTX-M-15_-producing ST8503 *E. coli* in the studied isolates*.*

Within the last several years, multiple studies across the world have reported rise of a new pandemic fluoroquinolones-resistant clonal group, *E. coli* ST1193. In this study, B2-ST1193 was the second most prevalent ST (10.3%). We also discerned that all of the B_2_-ST1193 isolates were resistant to ciprofloxacin and harboured *bla*_CTX-M-15_. Moreover, ST1193 has recently emerged from ST14CC as an important cause of extraintestinal disease in humans [46]. This is similar to some previous observations for different populations, such as community, hospital, and KTPs from which UPEC isolates had been derived in Switzerland [14], Iran [47], and Germany [39].

ST 38, on the other hand, has been identified as a hybrid of UPEC and enteroaggregative *E. coli* (EAEC) that is commonly associated with UTIs. In this study, ST410 was found in both groups of patients, one isolate, which co-produced *bla*_CTX-M-15_, *bla*_SHV_ and *bla*_TEM_ genes, belonged to phylogenetic group B_2_, while another ST38 *E. coli*, which had been previously reported in Germany, was associated with KTPs and belonged to phylogenetic group D [39]. Moreover, a report from Saudi Arabia stated that ST38 *E. coli* strains producing ESBL were one of the predominant ST in clinical settings [48].

We also noted that CC10 was the second predominant lineage disseminated among KTPs and non-KTPs. All members of CC10 were MDR and ESBL producer. They could also carry the *bla*_CTX-M_ gene: of them, ST744 and ST617 carry *bla*_CTX-M-55_ gene. Previous studies have also identified the association between members of CC10 and KTPs and hospital-acquired UTI [34, 39].

ST648 is one of the most successful and persistent clones and is also the most predominant genotype in CC648. In line with previous reports in Iran [49], we found ST648 to be associated with KTPs, phylogenetic group B_2_, and *bla*_CTX-M-15_ production in *E. coli* isolates.

In contrast to previous works, which announced ST69 as the predominant ST among KTPs and other population, we rarely found this ST among the studied strains so that only one ST69 was reported [39]. However, as previously reported, the ST69 have been predominantly detected among KTPs [39]. Moreover, in a study conducted in Iran, ST69 was reported with different antimicrobial resistance patterns [47].

In the current study, B_2_-ST405 was isolated from a KTP and considered as high-risk epidemic clone owing to its ability to co-produce *bla*_CTX-M-15_, *bla*_TEM_, *fimH*, *PAI*, *sfa*, *iut*, and *papG1* genes and disseminate β-lactamases. ST405 had not been already reported from KTPs. So, we reported for the first time the emergence of ST405 in KTPs [34, 39].

The main finding of this study was that the ST8503 and ST405 clones had not been previously reported among KTPs. this could be indicative of the fact that the spread of these ESBL-producing ST is an alarm about infection control policies. Thus, it is essential to recognize the clonal relatedness in the KTPs to amplify the surveillance and infection control system in clinical setting.

Another issue investigated in the current work was detection of isolates belonging to CC131, CC648, CC69, and CC 14 (international epidemic UPEC clones rapidly transmittable) in Iran.

The present research had limitations. The first, UPEC isolates was obtained only from there hospitals; thus the results should be interpreted with caution. Second, the impossibility of using typing methods such as pulsed-field gel electrophoresis (PFGE).

In conclusion, in this work, which was the first report on phylogenetic characterization and MLST analysis of UPEC isolates obtained from KTPs in Iran, we revealed significant differences between KTPs and non-KTPs regarding the phylogroups C and *PAI* gene of the UPEC isolates. We also observed differences between UPEC isolates obtained from the two studied group based on factors, such as the patients’ phylogenetic background (phylogroups and MLST analysis), their antibiotic resistance, and VF scores.

Moreover, based on MLST analysis, a relatively high diversity was observed in UPEC isolates obtained from KTPs and non-KTPs. Eighteen ST and nine clonal complexes were also identified. Pandemic UPEC clones including CC131 were also found to be the most prevalent ST types and clones. Further, ST 8503 was reported for the first time in KTPs. These results suggested regular studies on characterization of UPEC isolates among KTPs to help clinicians prescribe the most appropriate antibiotic and prevent further development of antimicrobial drug resistance.

## Supplementary Information

**Additional file 1: Figure S1.** (A) Agarose gel electrophoresis of PCR products for *sfa* (1177 bp) gene. M: 100 bp DNA size marker. (B) Agarose gel electrophoresis of PCR products for *hlyA* (1177 bp) gene. M: 100 bp DNA size marker. (C) Agarose gel electrophoresis of PCR products for *papG III* (258 bp) gene. M: 100 bp DNA size marker. (D) Agarose gel electrophoresis of PCR products for *iutA* (300 bp) gene. M: 100 bp DNA size marker. (E) Agarose gel electrophoresis of PCR products for *papG1* (461 bp) gene. M: 100 bp DNA size marker. (F) Agarose gel electrophoresis of PCR products for *pai* (930 bp) gene. M: 100 bp DNA size marker. (G) Agarose gel electrophoresis of PCR products for *fimH* (508 bp) and (*afa* 750 bp) genes. M: 100 bp DNA size marker. **Figure S2.** Agarose gel electrophoresis of Quadruplex PCR profiles of Clermont phylo-typing method. D: phylogenetic group D; A: phylogenetic group A; B_2_: phylogenetic group B_2_; Un: Unknown phylogenetic group M: 100 bp DNA size marker.

## Data Availability

Not applicable.

## Abbreviations

ESBL: Extended-spectrum β-lactamases
CLSI: Clinical and Laboratory Standards Institute
CCs: Clonal complex
DLV: Double-locus variants
DDST: Double-disk synergy test
KTP: Kidney transplant patients
ST: Sequence types
SLV: Single-locus variants
SD: Standard deviation
UPEC: Uropathogenic *Escherichia coli*
UTI: Urinary tract infections
MLST: Multilocus sequence typing
VFs: Virulence factors
