# Supplementary material for: Molecular epidemiology of blaCTX-M gene-producing uropathogenic Escherichia coli among Iranian kidney transplant patients: clonal dissemination of CC131 and CC10
Source: Ann Clin Microbiol Antimicrob. 2021 Sep 8;20:65. doi: 10.1186/s12941-021-00470-7 (PMC8424993; doi:10.1186/s12941-021-00470-7)
Supplement: Supplementary file 1 — Additional file 1: Figure S1. (A) Agarose gel electrophoresis of PCR products for sfa (1177 bp) gene. M: 100 bp DNA size marker. (B) Agarose gel electrophoresis of PCR products for hlyA (1177 bp) gene. M: 100 bp DNA size marker. (C) Agarose gel electrophoresis of PCR products for papG III (258 bp) gene. M: 100 bp DNA size marker. (D) Agarose gel electrophoresis of PCR products for iutA (300 bp) gene. M: 100 bp DNA size marker. (E) Agarose gel electrophoresis of PCR products for papG1 (461 bp) gene. M: 100 bp DNA size marker. (F) Agarose gel electrophoresis of PCR products for pai (930 bp) gene. M: 100 bp DNA size marker. (G) Agarose gel electrophoresis of PCR products for fimH (508 bp) and (afa 750 bp) genes. M: 100 bp DNA size marker. Figure S2. Agarose gel electrophoresis of Quadruplex PCR profiles of Clermont phylo-typing method. D: phylogenetic group D; A: phylogenetic group A; B2: phylogenetic group B2; Un: Unknown phylogenetic group M: 100 bp DNA size marker. [file 12941_2021_470_MOESM1_ESM.docx]

**Additional file 1:**


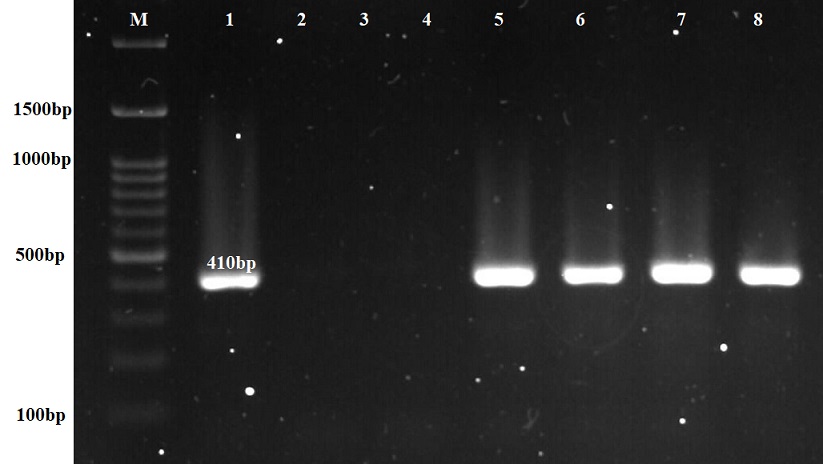


Figure S1 (A): Agarose gel electrophoresis of PCR products for *sfa* (1177bp) gene. M: 100 bp DNA size marker


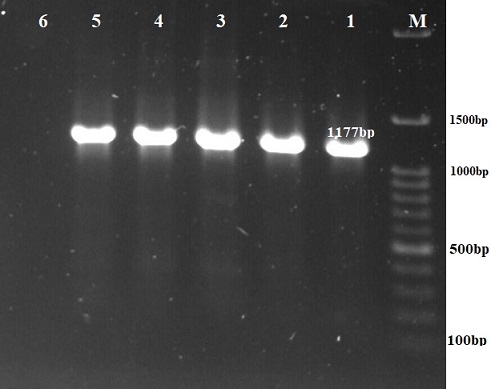


Figure S1 (B): Agarose gel electrophoresis of PCR products for *hlyA* (1177bp) gene. M: 100 bp DNA size marker


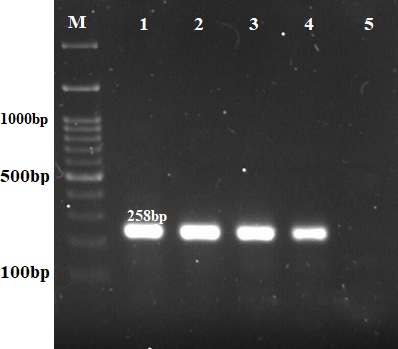


Figure S1 (C): Agarose gel electrophoresis of PCR products for *papG III* (258bp) gene. M: 100 bp DNA size marker


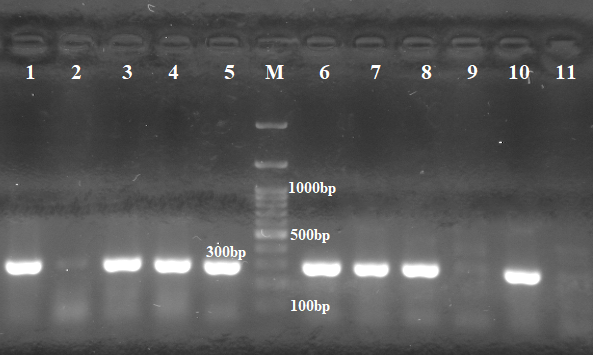


Figure S1 (D): Agarose gel electrophoresis of PCR products for *iutA* (300bp) gene. M: 100 bp DNA size marker


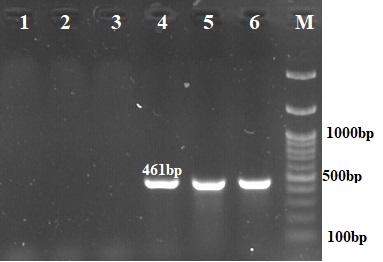


Figure S1 (E): Agarose gel electrophoresis of PCR products for *papG1* (461bp) gene. M: 100 bp DNA size marker


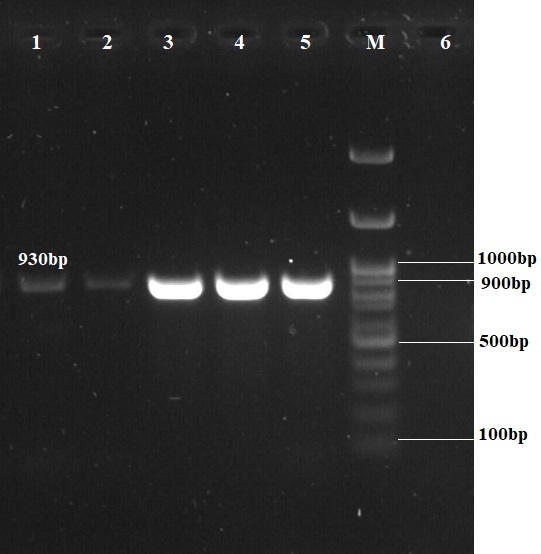


Figure S1 (F): Agarose gel electrophoresis of PCR products for *pai* (930bp) gene. M: 100 bp DNA size marker


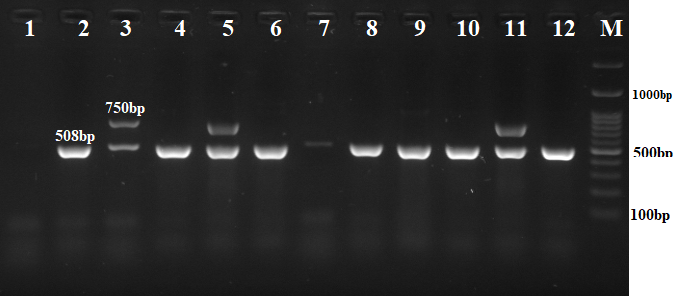


Figure S1 (G): Agarose gel electrophoresis of PCR products for *fimH* (508bp) and (*afa 750bp)* genes. M: 100 bp DNA size marker


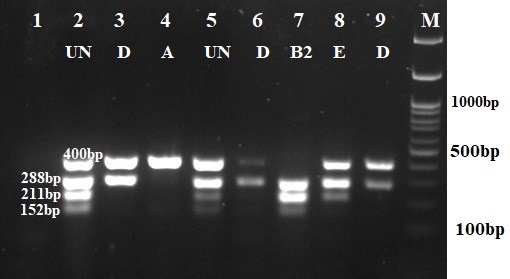


Figure S2: Agarose gel electrophoresis of Quadruplex PCR profiles of Clermont phylo-typing method. D: phylogenetic group D; A: phylogenetic group A; B_2_: phylogenetic group B_2_; Un: Unknown phylogenetic group M: 100 bp DNA size marker
